# Supplementary material for: Nano-Theranostics Constructed from Terpyridine-Modified Pillar [5]arene-Based Supramolecular Amphiphile and Its Application in Both Cell Imaging and Cancer Therapy
Source: Molecules. 2022 Sep 29;27(19):6428. doi: 10.3390/molecules27196428 (PMC9572324; doi:10.3390/molecules27196428)
Supplement: Supplementary file 1 [file molecules-27-06428-s001.zip › molecules-1905300-supplementary.pdf]

Nano-theranostics constructed from terpyridine modified pillar[5]arene based supramolecular amphiphile and its application in both cell imaging and cancer therapy

Youjun Zhou,[#,1,2] Lu Yang,[#,1] Longtao Ma,[1,2] Ying Han,[1] Chao-Guo Yan,*[,1] and Yong Yao*[,2]

[1]*School of Chemistry and Chemical Engineering, Yangzhou University, Yangzhou, Jiangsu, 225001, P.R. China*
[2]*School of Chemistry and Chemical Engineering, Nantong University, Nantong, Jiangsu, 22 6019, P.R. China*

*Correspongding author. E-mail address: cgyan@yzu.edu.cn; yaoyong1986@ntu.edu.cn
[#]Y. Zhou and L. Yang contributed equally to this work.

Supporting Information

1	Materials and methods	S2
2	Syntheses of **TP5**	S3
3	Synthesis of guest molecule	S8
4	Synthesis of **TP5**/Zn	S9
5	Host-guest interaction	S10
6	Controlled drug release	S11

1. Materials and methods

All reagents were commercially available and used as supplied without further purification. Solvents were either employed as purchased or dried according to procedures described in the literature. ^{1}H or ^{13}C NMR spectra were recorded with a Bruker Avance DMX 400 spectrophotometer with use of the deuterated solvent as the lock and the residual solvent or TMS as the internal reference. Solid-state nuclear magnetic resonance (NMR) spectra were recorded on a BRUKER 400WB AVANCE III spectrometer. Scanning electron microscopy (SEM) investigation was carried out on a JEOL 6390LV instrument. Transmission electron microscopy (TEM) images were obtained using a JEM-1200EX instrument with an accelerating voltage of 80 kV. UV−Vis spectroscopy was measured on a Shimadzu UV-2501 PC UV−Vis spectrometer.

2. **Synthesis of terpyridine-modified-pillar[5]arene**

2.1 **Synthesis of BrP5**[S1]

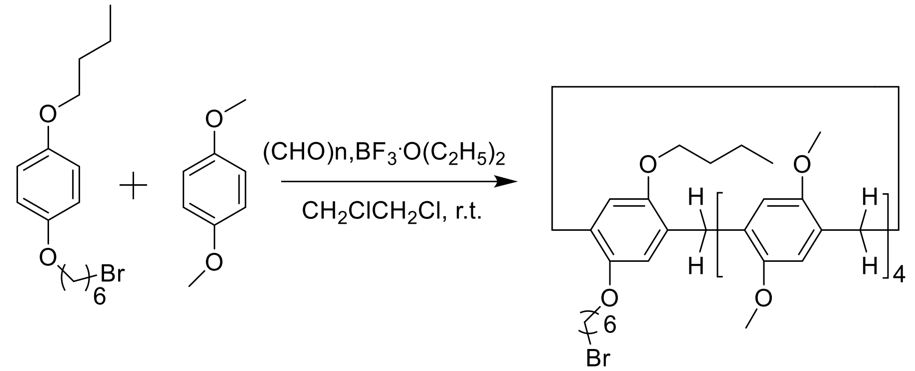

4C

BrP5

Scheme S1. synthetic route to **BrP5**.

4C monomer (20.0 mmol), p-dimethyl ether (13.8 g, 100.0 mmol) and paraformaldehyde (3.6 g) were added in a 500 mL round-bottom flask, stirring at room temperature and drop boron trifluoride diethyl ether (14.2 ml), and the reaction progress was detected by TLC. After the reaction was completed, methanol (40 mL) and dichloromethane (80 mL) were added to quench the reaction, and then saturated sodium bicarbonate solution was added for extraction. The lower layer liquid was collected, concentrated, and subjected to column chromatography (petroleum ether: dichloromethane: ethyl acetate = 100: 75: 1), and finally **BrP5** was obtained.

2.2 **Synthesis of terpyridine**[S2]

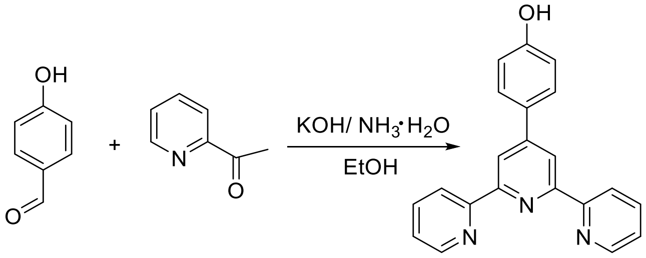

Scheme S2. synthetic route to **terpyridine**.

4-hydroxybenzaldehyde (3.05 g, 25.0 mmol), potassium hydroxide (4.20 g, 75.0 mmol), 2-acetylpyridine (6.05 g, 50.0 mmol) and ethanol (150 mL) were added in a two-necked flask. After stirring for 1 h, ammonium hydroxide (60 mL, 25%) was slowly added dropwise to the above reaction solution. The obtained solution was stirred under the protection of nitrogen at 50 °C for 12 h. The solvent was evaporated under vacuum and separated by thin layer chromatography (dichloromethane: methanol = 20: 1) to finally obtain a white solid (3.09 g, 38%).

2. 3 Synthesis of **TP5**

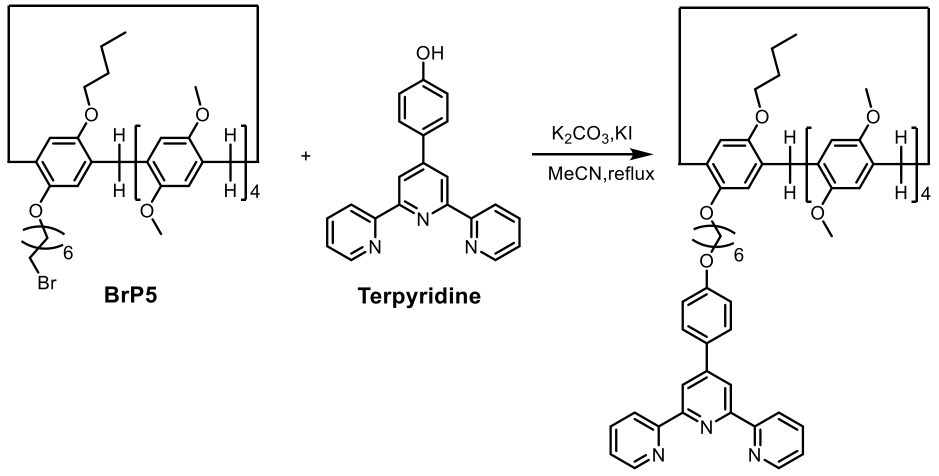

Scheme S3. Synthetic route of **TP5**.

Monobromo-substituted pillar[5]arene (**BrP5**) (0.43 mmol), 4-([2,2':6',2''-terpyridin]-4'-yl)phenol (**Terpyridine**) (0.14 g, 0.86 mmol), anhydrous potassium carbonate (0.1 g, 0.72 mmol), potassium iodide (trave), and dry acetonitrile were added in a 100 mL single-necked flask under N_2. The obtained solution was refluxed for 24 h and the reaction progress was detected by TLC. After the reaction was completed, it was cooled to room temperature, and after concentrating, thin-layer chromatography (dichloromethane: methanol = 20: 1 to 15: 1) was used to obtain **TP5** with medium yield.

TP5: White solid, 72%, m.p.83-85°C; ^{1}H NMR (400 MHz, $CDCl_3$) δ 8.72-8.71 (m, 4H, ArH), 8.66 (d, J = 8.0 Hz, 2H, ArH), 7.87-7.82 (m, 4H, ArH), 7.32-7.30 (m, 2H, ArH), 7.00 (d, J = 8.4 Hz, 2H, ArH), 6.81-6.76 (m, 10H, ArH), 3.92 (t, J = 6.4 Hz, 2H, CH2), 3.86 (t, J = 6.4 Hz, 2H, CH2), 3.82 (t, J = 6.4 Hz, 2H, CH2), 3.78-3.77 (m, 10H, CH2), 3.65-3.62 (m, 24H, OCH3), 1.82-1.78 (m, 2H, CH2), 1.75-1.71 (m, 4H, CH2), 1.55-1.40 (m, 6H, CH2), 0.93 (t, J = 7.6 Hz, 3H, CH3); ^{1}3C NMR (100 MHz, $CDCl_3$) δ 162.8, 159.0, 158.5, 153.4, 153.4, 152.7, 152.6, 152.4, 151.7, 139.5, 133.1, 131.1, 131.1, 131.0, 131.0, 130.9, 130.8, 130.8, 130.7, 126.4, 124.0, 120.9, 117.6, 117.5, 116.8, 116.7, 116.6, 116.6, 80.0, 79.8, 79.6, 71.0, 70.8, 70.6, 58.5, 58.4, 58.4, 58.3, 34.5, 32.4, 32.4, 32.3, 32.2, 31.8, 28.8, 28.6, 22.1, 16.6; MS (m/z): HRMS (ESI) Calcd. for C74H80N3O11 ($[M + H]^+$): 1186.5793, found: 1186.5783.

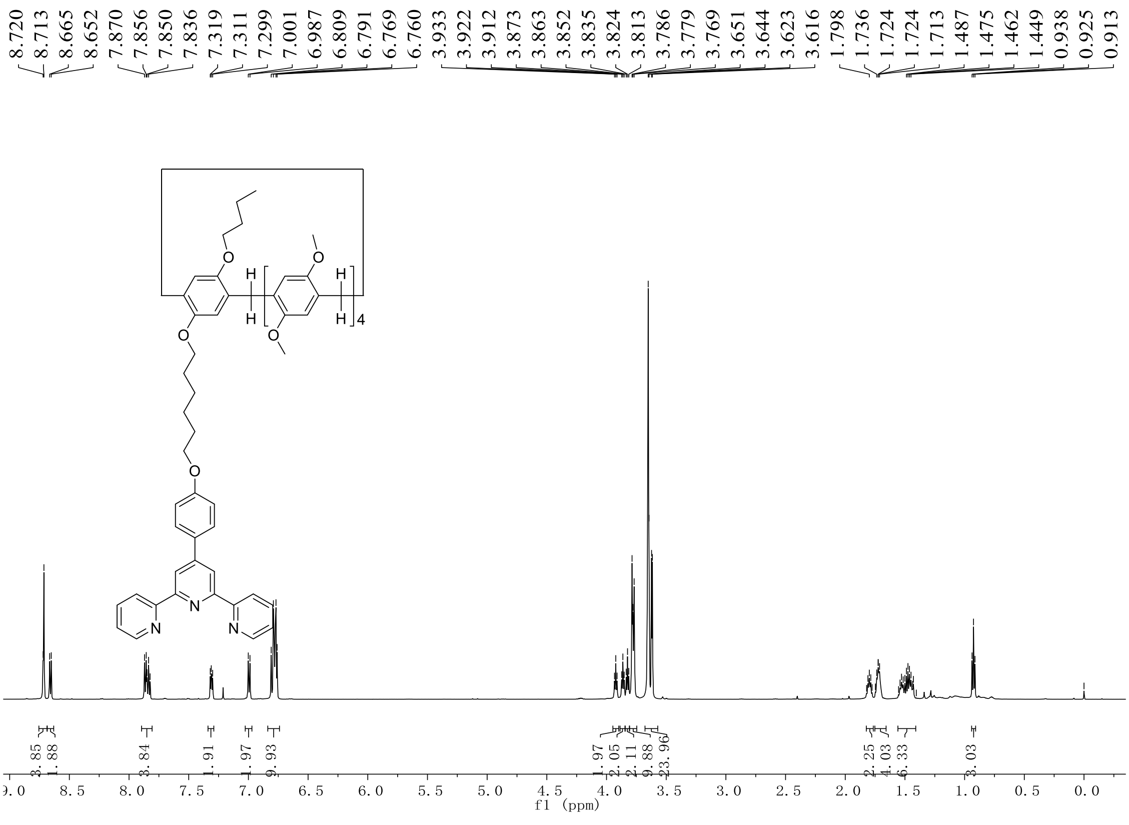

Figure S1. ^{1}H NMR spectrum of **TP5**（400 MHz, $CDCl_3$, 298K）

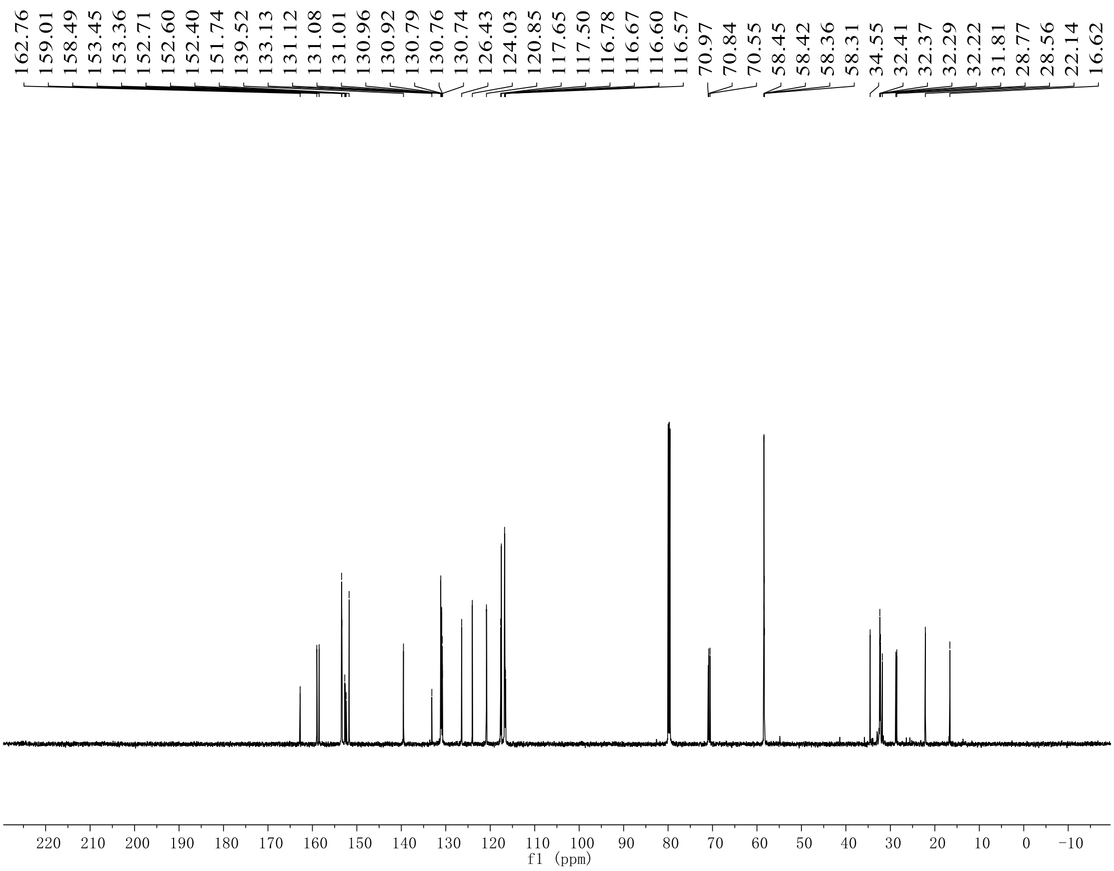

Figure S2. ^{13}C NMR spectrum of **TP5**（100 MHz, $CDCl_3$, 298K）

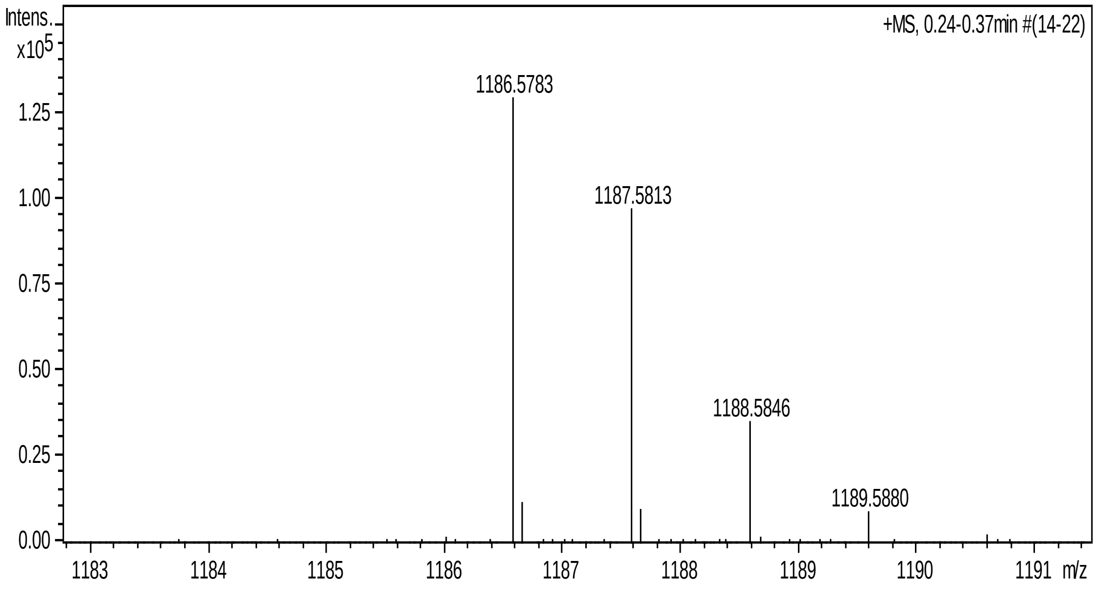

Figure S3. Mass spectra of **TP5.**

Table S1 Information of crystal data for **TP5**

Phase	**TP5**
Empirical formula	C74H80N3O11
Formula weight	1171.56
Temperature/K	296(2)
Wavelength/ Å	0.71073
Crystal system	Triclinic
Space group	P-1
a /Å	11.0701(11)
b / Å	11.8863(13)
c / Å	28.924(3)
α (°)	80.753(3)
β (°)	88.486(3)
γ (°)	67.891(3)
V (Å 3)	3477.9(6)
Z	2
Calculated density ($g \cdot cm^{-3}$)	1.295
Absorption coefficient (mm^{-1})	0.233
F(000)	1432
θ range / (°)	0.714 to 25.997
Limiting indices	-13<=h<=13, -14<=k<=14,<br>-35<=l<=35
Reflections collected/unique	46684 / 13564 [R(int) = 0.1630]
Completeness to theta	99.0 %
Max. and min. transmission	0.954 and 0.946
Refinement method	Full-matrix least-squares on F^2
Data/restraints/parameters	13564 / 0 / 856
Goodness-of-fit on F2	1.012
Final R indices[I>2sigma(I)]	R1 = 0.1069, wR2 = 0.2241

R indices (all data)	R1 = 0.2752, wR2 = 0.2923
Largest diff. peak and hole /(e ·Å-3)	0.853 and -0.745

3. Synthesis of guest molecule PM

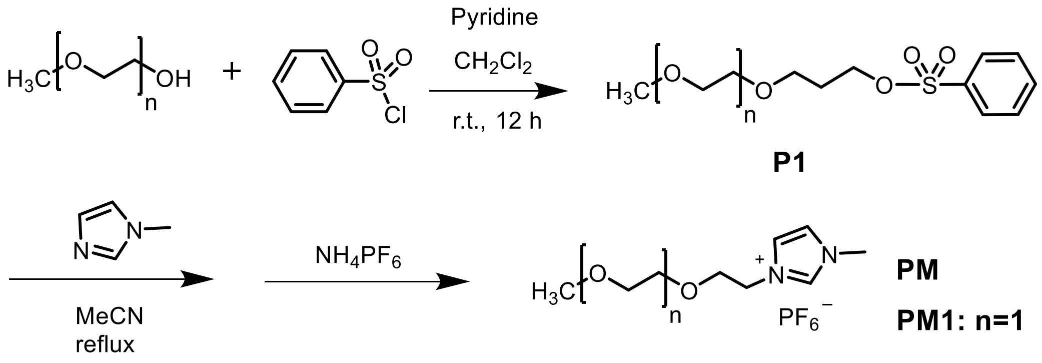

Scheme S4. Synthetic route of **PMs**.

Monomethoxypolyethylene glycol (M.W. = 750) (14.0 g, 40.0 mmol), pyridine (3.5 g, 44.0 mmol) and dichloromethane (30 mL) were added in a 100 mL single-neck flask and placed in an ice bath. The obtained solution was stirred in the ice bath and cooled to 0 °C. Benzenesulfonyl chloride (7.8 g, 44.0 mmol) was added dropwise over 30 min, removed from the ice bath, and stirred at room temperature for 12 h. The white precipitate was removed by filtration, and the filtrate was washed twice with brine, dried with anhydrous magnesium sulfate, and dried under vacuum to obtain the oily substance **P1**.

P1 (4.3 g, 10.0 mmol) and acetonitrile (10 mL) were added in a 50 mL single-necked flask, cooled to 0 °C in an ice bath, and 1-methylimidazole (0.9 g, 11.0 mmol) was added dropwise with stirring. The obtained solution was refluxed for 24 h, washed with ethyl acetate (10 mL × 3), and the resulting oil was dissolved in acetonitrile solution, added 1.2 equivalents of NH_4PF_6, and refluxed for 3 h. Cooling, diatomaceous earth filtration, vacuum drying to obtain oily liquid **PM**. The monomethoxy polyethylene glycol was replaced with 2-chloroethyl methyl

ether, and **PM1** was prepared according to the above synthesis steps. **PM1** was used as a template molecule to participate in compound characterization.

PM1: Yellow liquid, 78%; ^{1}H NMR (400 MHz, $CDCl_3$) δ 9.81 (s, 1H, ArH), 7.70 (d, J = 1.6 Hz, 2H, ArH), 4.62-4.57 (m, 2H, CH2), 3.80 (t, J = 4.8, 3H, CH3), 3.37 (d, J = 1.7 Hz, 3H, CH3), 3.26 (s, 2H, CH2); MS (m/z): HRMS (ESI) Calcd. for C7H13N2O (M^+): 141.1022, found: 141.1027.

4. Synthesis of TP5/Zn

TP5 (0.12 g, 0.1 mmol), $ZnCl_2$ (0.02 g, 0.12 mmol), and ethanol (40 mL) were added to a 100 mL round bottom flask, heated to reflux for 2 h, cooled to room temperature, recrystallized, and filtered (rinsed 3 times with ethanol) to obtain a light-yellow solid.

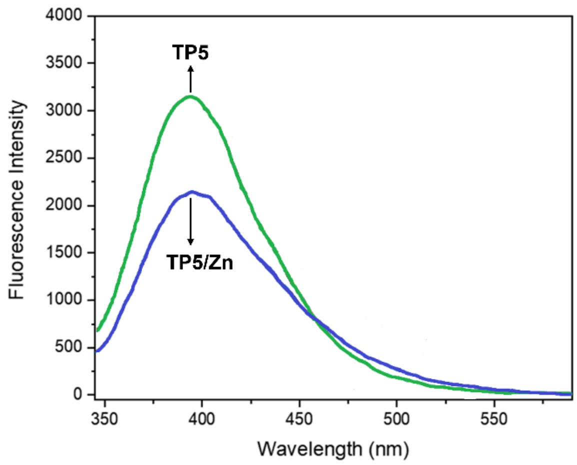

Figure S4. Fluorescence spectra of **TP5** and **TP5**/Zn.

5. Host-guest interaction

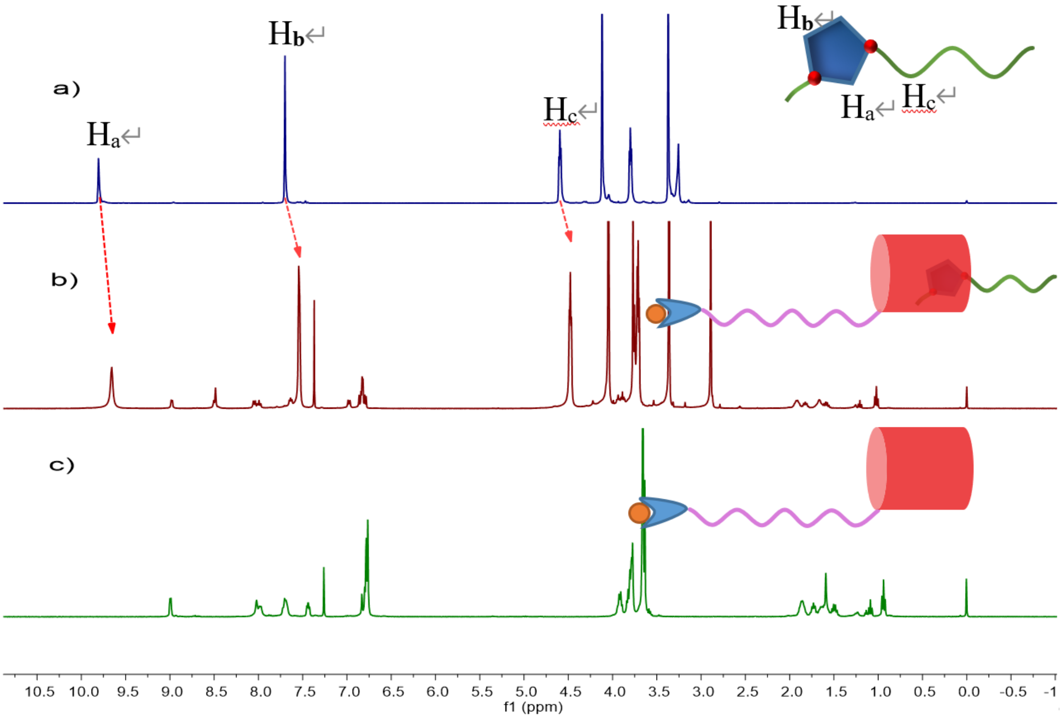

Figure S5. Partial ^{1}H NMR ($CDCl_3$, 400 MHz, R.T.) of: a) **PM1**(0.1mM); b) **PM1**(0.1mM) and **TP5**/Zn (0.1mM); c) **TP5**/Zn (0.1mM).

a)	Ha	Hb	Hc
δ	9.806	7.700	4.596
b)	Ha	Hb	Hc
δ	9.658	7.545	4.477
Δ δ	-0.148	-0.155	-0.119

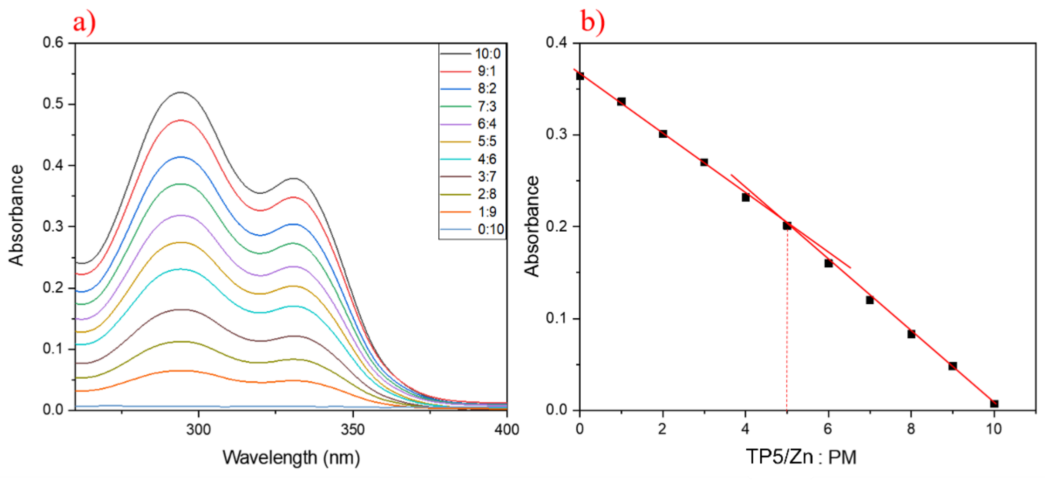

Figure S6. a) [**TP5**/Zn] + [**PM1**] = 2×10^{-5} M, UV-Vis spectra of **TP5**/Zn and **PM1**with different ratio； b) The absorbance at 330 nm of **TP5**/Zn and **PM1**with different ratio.

6. Controlled drug release

6.1 DOX loaded

Firstly, 0.0066 g of **TP5**/Zn was dissolved with 1 mL DMSO to obtain the solution with a concentration of 1 × 10^{-3} mol/L. Then 50 μL solution was added into 5mL-volumetric flask and added water to 5mL to obtain the aqueous solution with concentration 10^{-4} mol/L. A proper amount of PM solution was added into the aqueous solution of **TP5**/Zn, and the ratio of **TP5**/Zn to PM was 1:1.2. The ultrasound was conducted at room temperature for 20 min, then the same amount of DOX solution was added, and the ultrasound was continued for 20 min. The mixed solution of **TP5**/Zn/DOX was dialyzed in ultrapure water for several times, and the uncoated DOX and organic solvent DMSO were removed to prepare the aqueous solution of **TP5**/Zn/DOX nanoparticles.

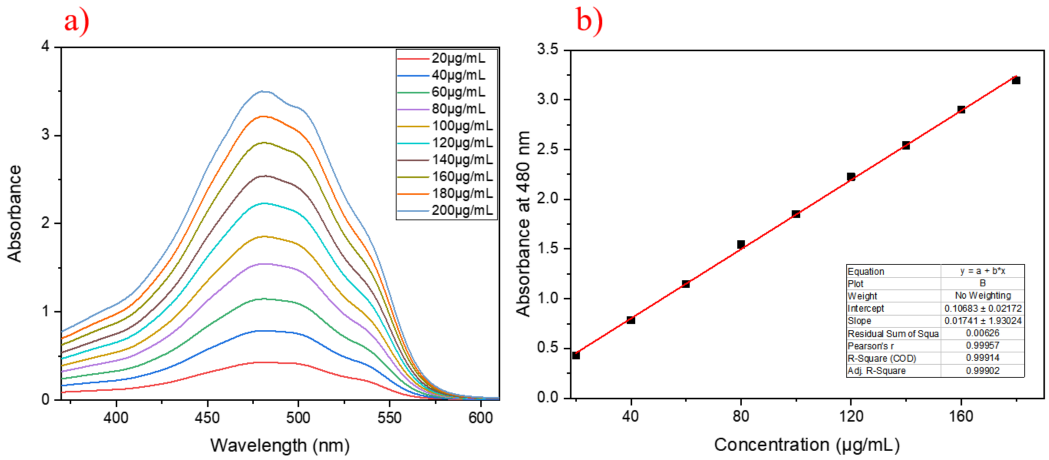

Figure S7. (a) UV-Vis absorption spectra (H_2O, RT) of different concentrations of Dox. (b) Absorbance intensity at 480 nm as a function of the concentration of Dox in solution.

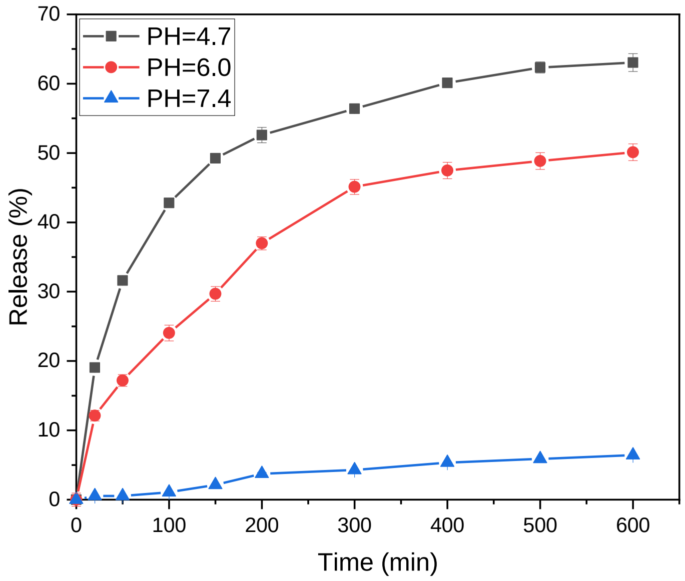

Figure S8. Drug release curves of **TP5/Zn/PM**-based vesicle in PBS solutions of different pH values at 37°C.

Reference

S1. Yao, Y.; Li, J.; Dai, J.; Chi, X.; Xue, M. A water-soluble pillar[6]arene: synthesis, host–guest chemistry, controllable self-assembly, and application in controlled release. *RSC Adv.* **2014**, *4*, 9039-9043.

S2. Shi, B.; Jie, K.; Zhou, Y.; Xia, D.; Yao, Y. Formation of fluorescent supramolecular polymeric assemblies via orthogonal pillar[5]arene-based molecular recognition and metal ion coordination. *Chem. Commun.*, **2015**, *51*, 4503-4506.
